# Supplementary material for: The East London Parkinson’s disease project – a case-control study of Parkinson’s Disease in a diverse population
Source: NPJ Parkinsons Dis. 2025 Jun 17;11:172. doi: 10.1038/s41531-025-01031-x (PMC12174313; doi:10.1038/s41531-025-01031-x)
Supplement: Supplementary file 1 — Supplementary files [file 41531_2025_1031_MOESM1_ESM.pdf]

Supplementary Table 1

	White n=96	South Asian n=85	Black n=24	White vs South Asian		White vs Black		Black vs South Asian	
				Uni	Multi	Uni	Multi	Uni	Multi
				p, t(dof)	p	p, t	p	p, t(dof)	p
**Smell test (median ± IQR)**	2.0 ± 1.0	2.0 ± 3.0	1.0 ± 1.0	0.911[e], 872.5	0.917[f], 0.986[g]	0.018[e], 233.5	0.006[f], 0.066[g]	0.037[e], -1.2	0.006[f], 0.119[g]
**HADS Depression (mean ± SD)**	6.3 ± 3.8	8.5 ± 5.2	6.8 ± 4.3	0.019[a], 5.5	0.052[f], **0.002[g]**	0.627[e], 560.5	0.055[f], 0.435[g]	0.245[d], 0.1(67)	0.007[f], 0.19[g]
**HADS Anxiety (median ± IQR)**	6.0 ± 6.0	8.0 ± 9.0	7.0 ± 6.0	0.077[a], 3.1	0.152[f], 0.026[g]	0.482[e], 539.5	0.061[f], 0.517[g]	0.514[e], 385.0	0.021[f], 0.281[g]
**RBDSQ (median ± IQR)**	3.0 ± 3.5	4.5 ± 5.8	7.0 ± 2.0	0.177[e], 897.0	0.297[f], 0.050[g]	0.035[e], 166.5	0.831[f], 0.013[g]	0.290[e], 279.5	0.923[f], 0.043[g]
**PDSS2 (mean ± SD)**	17.1 ± 10.2	23.1 ± 11.7	19 ± 9.3	0.008[e], 748.0	0.049[f], **0.003[g]**	0.464[e], 295.5	0.177[f], 0.495[g]	0.291[d], -1.1(49)	0.018[f], 0.187[g]
**ESS (median ± IQR)**	8.5 ± 8.0	11.0 ± 10.2	8.0 ± 9.2	0.073[a], 3.2	0.108[f], 0.009[g]	0.743[e], 987.0	0.137[f], 0.595[g]	0.475[e], 832.0	0.022[f], 0.362[g]
**SCOPA-AUT (mean ± SD)**	18.1 ± 9.6	17.5 ± 10.7	16.9 ± 12.8	0.776[d], 0.3(83)	0.541[f], 0.491[g]	0.456[e], 250.5	0.263[f], 0.654[g]	0.533[e], 143.5	0.291[f], 0.973[g]
**EQ5D5L VAS (mean ± SD)**	63.7 ± 16.7	53.3 ± 21.8	60.4 ± 22.0	**0.001[e], 5116.0**	0.073[f], 0.024[g]	0.740[e], 1129.5	**0.001[f]**, 0.335[g]	0.171[d], 1.4(106)	0.055[f], 0.682[g]
**EQ5D5L index (mean ± SD)**	0.5 ± 0.3	0.4 ± 0.3	0.4 ± 0.3	**<0.001[a], 12.2**	0.012[f], 0.005[g]	0.359[e], 1228.0	**0.002[f]**, 0.144[g]	0.293[d], 1.1(106)	0.15[f], 0.571[g]
**EQ5L5L pain (mean ± SD)**	2.5 ± 1.1	2.7 ± 1.1	2.5 ± 1.2	0.123[e], 3515.5	0.121[g]	0.855[e], 1066.0	0.849[g]	0.511[e], 1063.0	0.437[g]
**IMD decile (median ± IQR)**	4.0 ± 2.0	3.0 ± 1.0	3.0 ± 2.0	0.022[a], 5.3	0.037[k], 0.050[l]	**0.003[a], 8.73**	**<0.001[k], 0.003[l]**	0.101[e], 611.5	0.023[k], 0.016[l]

Supplementary Table 1. Clinical characteristics of patients with Parkinson's disease in the East London Parkinson's Disease project. d - two-sided T-test; e - Mann-Whitney U test; f - Logistic regression, adjusted for disease duration; g - Logistic regression, adjusted for disease duration, age and gender; k - Logistic regression, adjusted for gender; l - Logistic regression, adjusted for gender, age and years of education. SD - standard deviation; IQR - interquartile range; t - T statistic; dof - degrees of freedom; bold - p<0.004 (Bonferroni-adjusted for multiple comparisons).

Supplementary Table 2

	White n=52	South Asian n=45	Black n=8	White vs South Asian	White vs Black	Black vs South Asian	Black vs South Asian vs Black
				Uni	Uni	Uni	Uni
				p, t(dof)	p, t	p, t(dof)	p, t(dof)
**MERQ-PD-B Pesticides (n, %)**	2, 2%	0, 0%	0, 0%	-	-	-	0.613[c], 1.0
**MERQ-PD-B Head injury (n, %)**	9, 17%	5, 11%	1, 11%	-	-	-	0.658[c], 0.8
**MERQ-PD-B Caffeine previous consumption (n, %)**	51, 96%	41, 91%	9, 100%	-	-	-	0.568[c], 1.1
**MERQ-PD-B Caffeine previous consumption (mean ± SD)**	4.1 ± 2.3	3.1 ± 1.9	2.4 ± 0.8	0.026[e], 1445.5	0.088[e], 311.0	0.166[e], 144.5	-
**MERQ-PD-B Caffeine current consumption (n, %)**	47, 89%	37, 82%	7, 78%	-	-	-	0.547[c], 1.2
**MERQ-PD-B Caffeine current consumption (mean ± SD)**	2.8 ± 2.3	2.1 ± 1.5	1.4 ± 1.2	0.331[a], 0.9	0.101[e], 302.0	0.166[e], 0.6	-
**MERQ-PD-B Alcohol previous consumption (n, %)**	36, 75%	7, 17%	6, 67%				**<0.001[c], 29.9**
**MERQ-PD-B Alcohol current consumption (n, %)**	27, 56%	1, 2%	5, 56%				**<0.001[c], 30.8**
**MERQ-PD-B Smoking previous (n, %)**	24, 45%	8, 18%	0, 0%				**0.003[c], 11.8**
**MERQ-PD-B Smoking current (n, %)**	4, 8%	1, 2%	0, 0%				0.479[c], 1.5

Supplementary Table 2. Exposure in patients with Parkinson's disease in the East London PD study. a - Kruskall Wallis test; c - Fisher's exact test; k - Logistic regression adjusted for gender; l - Logistic regression adjusted for gender, age, years of education. IMD - Index of Multiple Deprivation; SD - standard deviation; IQR - interquartile range; bold - p<0.004 (Bonferroni-adjusted for multiple comparisons).

Supplementary Table 3

Chosen by highest UPDRS Left vs Right

**BRAIN test**	**White n=71**	**South Asian n=50**	**Black n=13**	**White vs South Asian**		**White vs Black**		**Black vs South Asian**	
				Uni	Multi	Uni	Multi	Uni	Multi
				p, t(dof)	p	p, t	p	p, t(dof)	p
**KS, n (mean ± SD)**	36.5 ± 14.4	31.4 ± 12.5	32.2 ± 17.6	0.052[d], 2.0(115)	0.085[f], 0.491[g], 0.335[h], 0.505[j]	0.358[d], 0.9(80)	0.007[f], 0.348[g], 0.317[h], 0.385[j]	0.846[d], 0.2(59)	0.076[f], 0.722[g], 0.728[h], 0.747[j]
**AT, ms (median ± IQR)**	153.0 ± 90.1	194.5 ± 102.2	214.5 ± 118.7	**0.002[a], 7.4**	0.171[f], **<0.001[g], <0.001[h], <0.001[h]**	0.018[e], 261.0	0.697[f], 0.008[g], 0.012[h], 0.008[j]	0.591[e], 343.0	0.152[f], 0.896[g], 0.874[h], 0.910[j]
**IS, ms^2 (mean ± SD)**	18752.8 ± 19072.6	26332.1 ± 25727.1	22224.8 ± 13556.3	0.142[e], 1008.0	0.306[f], 0.070[g], 0.075[h], 0.062[j]	0.178[e], 275.0	0.521[f], 0.301[g], 0.218[h], 0.255[j]	0.72[e], 257.0	0.214[f], 0.854[g], 0.957[h], 0.871[j]

Chosen by lowest KS Left vs Right

**BRAIN test**	**PwP n=145**	**HC n=13**	**p-value**
**KS, n (mean ± SD)**	34.7 ± 14.6	46.3 ± 8.8	**<0.001[i]**, 15.8(152)
**AT, ms (mean ± SD)**	180.3 ± 88.9	150 ± 38.6	0.287[e], 1081.0
**IS, ms^2 (mean ± SD)**	22185.3 ± 22022.8	12145.7± 6590.8	0.414[a], 0.7

Supplementary Table 3. BRAIN test scores in the East London Parkinson's Disease project. a - Kruskal Wallis test, d - two-sided T-test; e - Mann-Whitney U test; f - Logistic regression, adjusted for disease duration; g - Logistic regression, adjusted for disease duration, age and gender; h - Logistic regression, adjusted for disease duration, age, gender, and ON-OFF status, i - Welch's ANNOVA test; j - Logistic regression, adjusted for disease duration, age, gender, and LEDD. KS - kinesia score, AT - akinaesia time; IS - incoordination score; SD - standard deviation; IQR - interquartile range; PwP – People with Parkinson's; HC – healthy controls; t - T statistic; dof - degrees of freedom, ms - millisecond, cm - centimetre ; bold - p<0.004 (Bonferroni-adjusted for multiple comparisons).

Supplementary Table 4

MoCA		White n=91	South Asian n=52	Black n=16	White vs South Asian	White vs Black	Black vs South Asian	Black vs South Asian vs Black
					Uni	Uni	Uni	Uni
					p, t(dof)	p, t	p, t(dof)	p, t(dof)
**PD normal cognition (n, %)**	MoCA > 25	50, 55%	14, 27%	4, 25%	**0.003**[b], 11.5	0.084[c], 4.9	0.688[c], 0.7	0.006[c], 14.4
**PD mild cognitive impairment (n, %)**	MoCA 19-25	33, 36%	27, 52%	10, 63%				
**PD dementia (n, %)**	MoCA < 19	8, 9%	11, 21%	2, 12%				
**PD normal cognition (n, %)**	MoCA > 25	50, 55%	14, 27%	4, 25%	**0.002**[b], 9.4	0.027[c], 4.9	0.879[c],0.1	**0.002[c], 12.9**
**PD cognitive impairment (n, %)**	MoCA <= 25	41, 45%	38, 73%	12, 75%				

Supplementary Table 4. Cognitive impairment in patients with Parkinson's Disease. b - Chi-squared test; c - Fisher's exact test; t - T statistic; dof - degrees of freedom; bold - p<0.004 (Bonferroni-adjusted for multiple comparisons).

Supplementary Figure 1

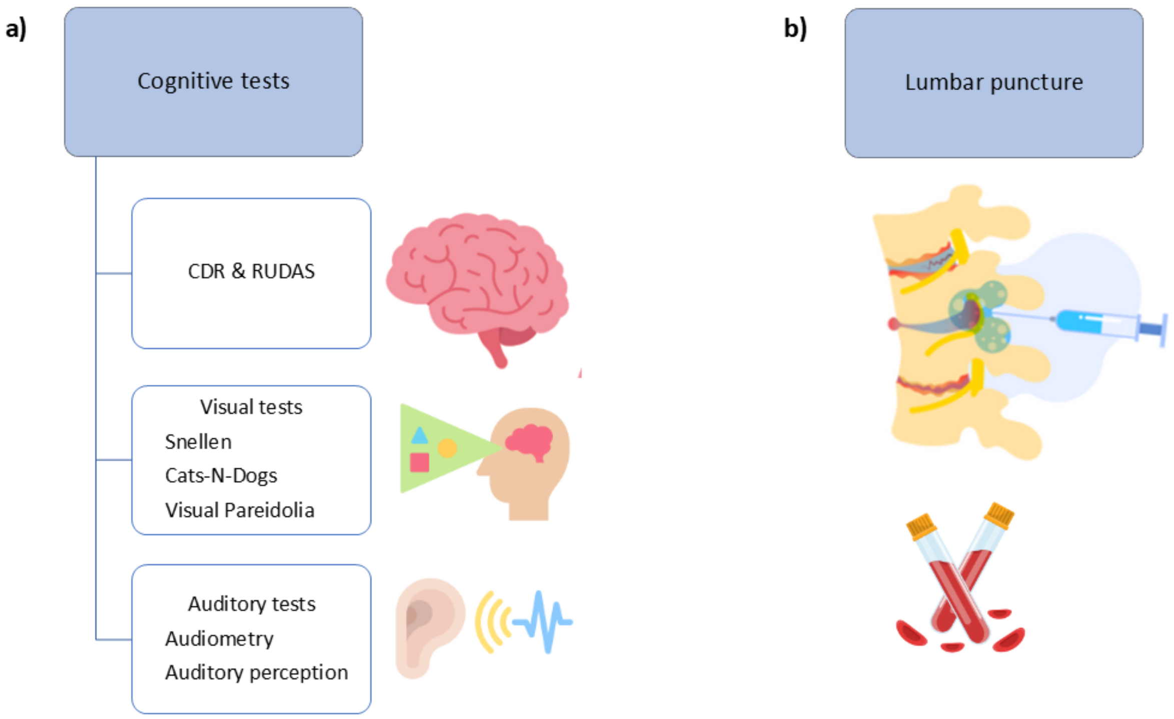

Supplementary Figure 1. a) Optional visit for the East London Parkinson's Disease study. CDR - Clinical Dementia Rating Scale[1]; RUDAS - Rowland Universal Dementia Assessment Scale[3]; Cats-N-Dogs – Cats-and-Dogs test[3]. b) Optional visit for the East London Parkinson's Disease Study consisting of a lumbar puncture.

Supplementary Note 1:

From 218 patients, 6 South Asian and 1 Arabic individuals were unable to complete the assessment for MoCA. They were excluded from the cognitive analysis due to a language barrier at the outset (3) and other reasons (2 could not follow commands due to advanced Parkinson's, and 2 had severe motor fluctuations). A total of 211 patients completed the MoCA assessment: 96 White, 79 South Asian, 24 Black patients and 12 from other backgrounds. Out of these 196 were performed in English, in some cases with a translator, and 15 directly in Bengali (validated translation). A further 44 patients were excluded, as although they were able to complete part of the assessment, the results were unreliable due to: evident language barriers during tests (15, 7%), low levels of literacy (9, 4%), severe tremor (4, 2%), blindness (4, 2%), severe dyskinesia (1, 0.4%), severe dystonia (1, 0.4%) and other reasons (10, 5%). The majority of the patients who had unreliable scores were from South Asian backgrounds (24, 61%). Out of 167 patients, 91 White, 52 South Asian, 16 Black and 8 other patients had reliable cognitive scores on the MoCA (n=159).

From 90 controls, 45 had MoCA assessments: 14 White, 30 South Asian, 1 other. Black controls did not have any available data. A further 6 scores in the controls were unreliable due to: language barrier (5, 11%) and other reasons (1, 2%).

Supplementary References:

1. O'Bryant SE, Waring SC, Cullum CM, Hall J, Lacritz L, Massman PJ, et al. Staging dementia using Clinical Dementia Rating Scale Sum of Boxes scores: a Texas Alzheimer's research consortium study. Arch Neurol. 2008 Aug;65(8):1091–5.
2. Storey JE, Rowland JTJ, Basic D, Conforti DA, Dickson HG. The Rowland Universal Dementia Assessment Scale (RUDAS): a multicultural cognitive assessment scale. Int Psychogeriatr. 2004 Mar;16(1):13–31.
3. Weil RS, Pappa K, Schade RN, Schrag AE, Bahrami B, Schwarzkopf DS, et al. The Cats-and-Dogs test: A tool to identify visuoperceptual deficits in Parkinson's disease. Mov Disord. 2017 Dec;32(12):1789–90.
